# Human astrovirus capsid protein releases a membrane lytic peptide upon trypsin maturation

**DOI:** 10.1128/jvi.00802-23

**Published:** 2023-07-28

**Authors:** Matthew Ykema, Kai Ye, Meng Xun, Justin Harper, Miguel A. Betancourt-Solis, Carlos F. Arias, James A. McNew, Yizhi Jane Tao

**Affiliations:** 1 Department of BioSciences, Rice University, Houston, Texas, USA; 2 Departamento de Genética del Desarrollo y Fisiología Molecular, Universidad Nacional Autónoma de México, Cuernavaca, Morelos, Mexico; University of Kentucky College of Medicine, Lexington, Kentucky, USA

**Keywords:** astrovirus, structural biology, liposome, proteases

## Abstract

The human astrovirus (HAstV) is a non-enveloped, single-stranded RNA virus that is a common cause of gastroenteritis. Most non-enveloped viruses use membrane disruption to deliver the viral genome into a host cell after virus uptake. The virus–host factors that allow for HAstV cell entry are currently unknown but thought to be associated with the host-protease-mediated viral maturation. Using *in vitro* liposome disruption analysis, we identified a trypsin-dependent lipid disruption activity in the capsid protein of HAstV serotype 8. This function was further localized to the P1 domain of the viral capsid core, which was both necessary and sufficient for membrane disruption. Site-directed mutagenesis identified a cluster of four trypsin cleavage sites necessary to retain the lipid disruption activity, which is likely attributed to a short stretch of sequence ending at arginine 313 based on mass spectrometry of liposome-associated peptides. The membrane disruption activity was conserved across several other HAstVs, including the emerging VA2 strain, and effective against a wide range of lipid identities. This work provides key functional insight into the protease maturation process essential to HAstV infectivity and presents a method to investigate membrane penetration by non-enveloped viruses *in vitro*.

**IMPORTANCE:**

Human astroviruses (HAstVs) are an understudied family of viruses that cause mild gastroenteritis but have recent cases associated with a more severe neural pathogenesis. Many important elements of the HAstV life cycle are not well understood, and further elucidating them can help understand the various forms of HAstV pathogenesis. In this study, we utilized an *in vitro* liposome-based assay to describe and characterize a previously unreported lipid disruption activity. This activity is dependent on the protease cleavage of key sites in HAstV capsid core and can be controlled by site-directed mutagenesis. Our group observed this activity in multiple strains of HAstV and in multiple lipid conditions, indicating this may be a conserved activity across the AstV family. The discovery of this function provides insight into HAstV cellular entry, pathogenesis, and a possible target for future therapeutics.

## INTRODUCTION

Astrovirus (AstV) is a non-enveloped, positive-sense single-stranded RNA virus. In humans, infections caused by the eight classical human astroviruses (HAstVs) often result in mild to severe gastroenteritis, with more severe symptoms seen in children and the elderly. A wider range of diseases is observed in non-human AstV infections, including viral-associated mink shaking syndrome, lethal encephalitis in bovine, and gout in broiler chickens (1 - 3). In addition to gastroenteritis, neural pathogenesis has been observed in humans infected with the emerging VA and MLB clades as well as immunocompromised individuals infected with classic HAstV strains (4 - 7). Phylogenetic analyses indicate that these emerging HAstV strains arose from recent animal–human host crossover events, resulting in human pathogenesis that mirrors those seen in other animal hosts (8). Understanding the properties of HAstVs and their infection mechanisms should provide further insights into viral pathogenesis and possible therapeutic solutions.

**Fig 1:**
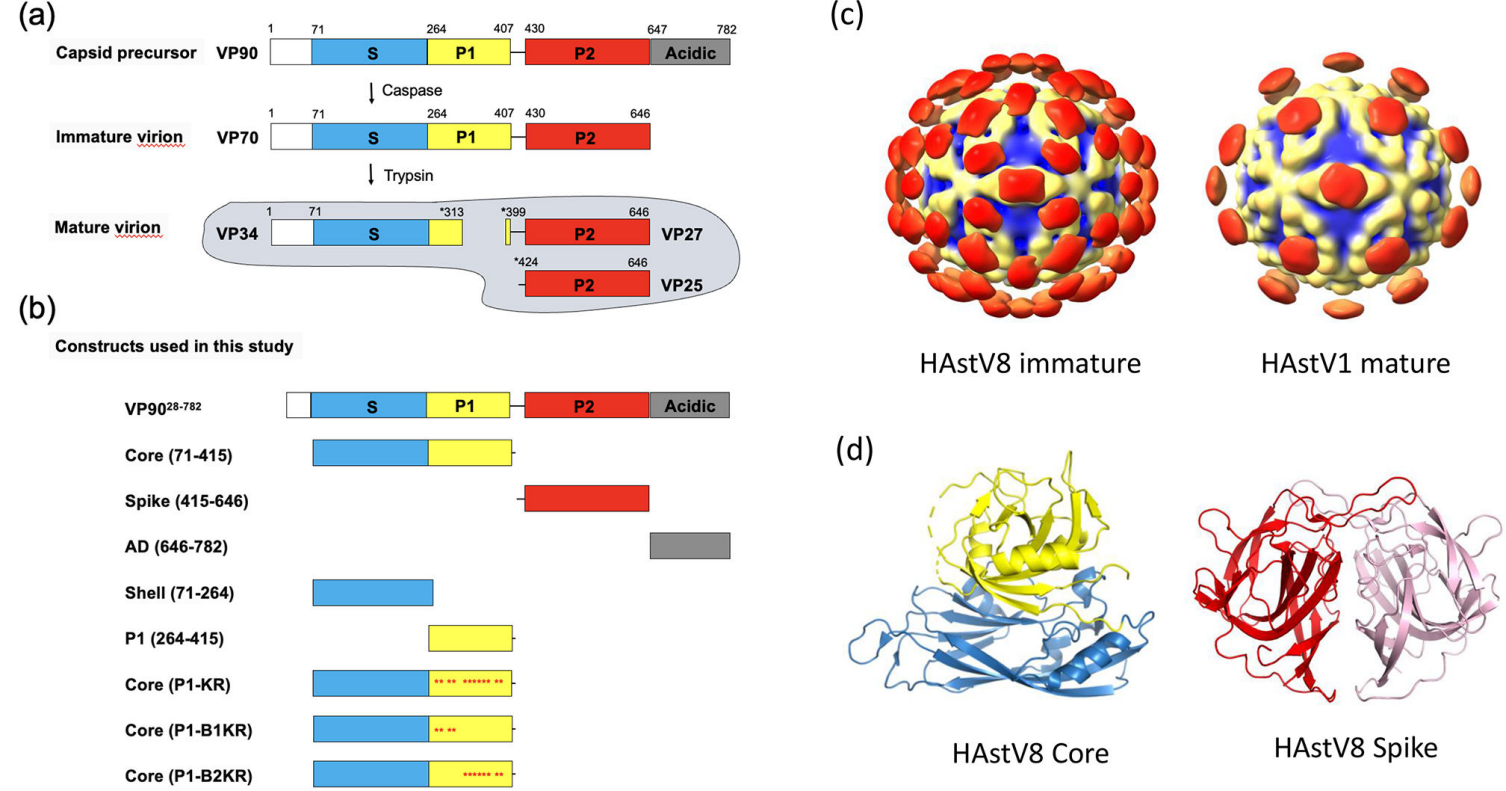
HAstV8 maturation process and constructs used in this study. (**a**) The maturation process of the HAstV8 capsid protein VP90 catalyzed by caspases and trypsin. (**b**) Recombinant protein constructs used in this study. Red asterisks indicate sites where amino acids recognized by trypsin (arginine and lysine) were mutated. (**c**) Cryo-EM maps of the immature HAstV8 VP70 and mature HAstV1 VP34/27/25 particles. Left, EMDB ID: EMD-5414. Right, EMDB ID: EMD-5413. (**d**) Crystal structures of the S-P1 core and P2 spike. Left, PDB ID: 5IBV. Right, PDB ID: 3QSQ. The S and P1 domains in the core are colored in blue and yellow, respectively. In the spike dimer, the two subunits are colored in different shades of red.

One of the key properties of HAstVs is the dependence on human host proteases to facilitate key stages of the viral life cycle. HAstVs express one structural polyprotein precursor that contains several structural domains: the N-terminal basic domain, the S inner core domain, the P1 outer core domain, the P2 spike domain, and the C-terminal acidic domain (AD) (9) (Fig. 1a). For the HAstV serotype 8 (HAstV8), a total of 180 copies of the structural polyprotein precursor VP90 form the membrane-associated precursor capsid. After VP90 particle assembly, the AD is cleaved off by host caspases. This results in the immature VP70 capsid that can be released from the infected cell. The VP70 capsid state is unable to efficiently infect new host cells, but it is susceptible to digestion by extracellular trypsin, which cleaves at multiple sites in the P1 domain (10). The resulting mature virus, VP34/27/25, has multiple log fold higher infectivity when compared to the immature VP70 virus (11, 12). The mature virus binds an as-yet unidentified host cell receptor and enters the cell by clathrin-mediated endocytosis (13). Upon entry, viral capsid uncoating is facilitated by the host factor PDIA4 through association with the HAstV spike domain (14). There are still several unknown elements involved in the HAstV infection process, such as capsid property changes induced by trypsin cleavage, the identity of the host cell receptor for viral entry, and the role of lipid membranes that the HAstV structural protein precursors associate with during assembly (15).

Our understanding of HAstVs has been enhanced by studies of the structure and assembly of the HAstV capsid protein. A preliminary map of the immature and mature virion has been solved by cryo-electron microscopy (EM) (Fig. 1c) (10). The immature VP70 state of the virion has a *T* = 3 icosahedral structure similar to hepatitis E virus, with 90 surface spikes formed by dimers of the P2 domain. Upon treatment with trypsin, there is a significant morphological change in the capsid resulting in the loss of 60 of the 90 capsid spikes (10). The 30 spikes along the icosahedral two-fold axes are retained, exposing a large amount of the P1 outer core domain. It is currently unknown how this significant change in capsid morphology affects the virus–host interactions, but it is currently hypothesized to involve changes in capsid functions and/or interactions with host receptors (13, 16).

Crystal structures have been solved for the full core domain (*S* + P1) and the P2 spike domain from multiple classic HAstV serotypes (Fig. 1a). The crystal structures of the HAstV1 and HAstV8 core domains reveal a highly conserved structure with a S inner core made of a jelly roll beta-barrel fold capped with a P1 outer core made of a squashed beta-barrel with multiple trypsin-sensitive sites exposed on surface (16, 17). The core domain provides structural cohesion for the capsid during protease maturation and antibodies targeting this domain do not neutralize viral infection, indicating the structural conservation is likely due to low evolutionary pressure (18, 19). The structures of the HAstV1, HAstV2, and HAstV8 P2 spikes reveal a globular domain dominated by beta sheets with an interwoven dimer interface (16, 20, 21). While the overall structural fold is conserved among the three serotypes, large sequence variations are observed in localized areas due to the presence of antigen recognition sites (19, 21, 22).

It has been well established in the literature that non-enveloped viruses have a wide range of mechanisms to breach host cellular membranes to allow for the delivery of viral genomes into a new host (23, 24). One candidate mechanism is the cleavage of viral proteins to release an active peptide or domain that is capable of membrane penetration. This can be caused by autocatalytic activity, such as the formation of the flock house virus gamma peptide, or cleavage by host proteases, such as the cleavage of rotavirus VP4 into VP5 and VP8 by trypsin (25, 26). To investigate the relationship between trypsin cleavage and membrane penetration by the HAstV capsid, our group set out to express the HAstV8 VP90 using a heterologous expression system to prevent mammalian protease maturation. Using HAstV8 VP90 proteins, individual domains, and mutant constructs, we discovered a trypsin-dependent lipid disruption activity located in the P1 domain that is maintained across a range of HAstV strains and lipid compositions. This is the first reporting of a trypsin-dependent membrane disruption activity by the HAstV capsid using an *in vitro* assay, and we hypothesize that this activity likely plays an important role in cell entry and pathogenesis.

## RESULTS

### Purification and validation of HAstV8 VP90 by heterologous expression in *Escherichia coli*

**Fig 2:**
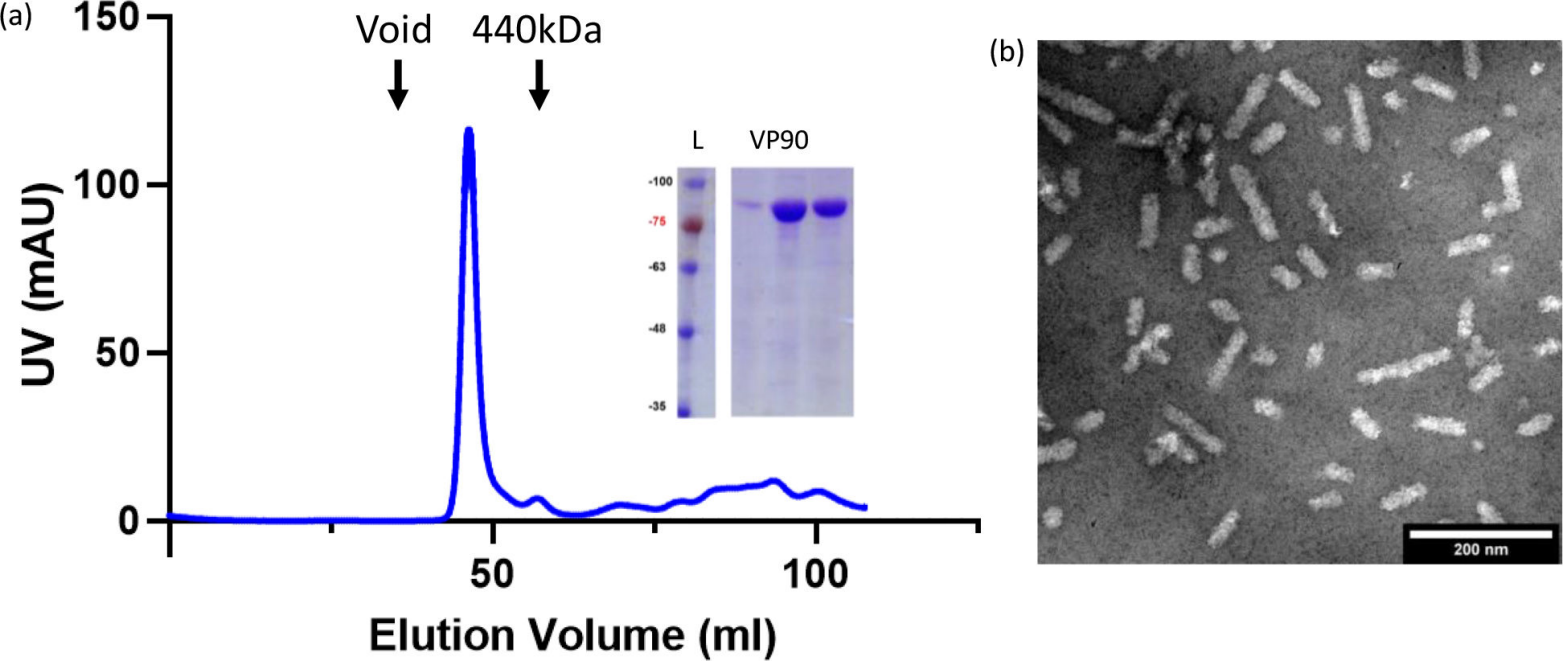
Expression and purification of the HAstV8 capsid protein VP90. (**a**) HAstV8 VP90^28-782^ purified by Superdex-200 (S200) size exclusion chromatography. The protein was eluted with a major peak at an elution volume of 46.4 mL, indicating a large molecular weight assembly. A black arrow marks the peak elution of the blue dextran molecular weight standard indicating void volume, while another arrow shows the peak elution of ferritin of 440 kD. Inset—protein size and stability were confirmed by SDS-PAGE. (**b**) TEM micrographs of HAstV8 VP90^28-782^. Rod-shaped particles were observed with a consistent diameter but variable lengths. Scale bar, 200 nm.

To characterize the HAstV8 protease maturation process *in vitro*, the VP90 precursor protein is required. Expression in mammalian systems may not be ideal considering that the presence of caspases would mature the capsid protein. It has also been observed that the inhibition of host caspases during HAstV8 infection resulted in VP90 aggregates that cannot be extracted from the insoluble cellular fractions (Dr. Carlos Arias, personal communication). Heterologous expression is an option to obtain soluble, intact VP90, and limited success has been shown previously in mammalian and insect cell-based systems (27, 28). *Escherichia coli* expression has been utilized previously for expressing HAstV8 capsid protein domains for structural characterization, but the full-length VP90 construct has not been tested in *E. coli* (17, 20). In this study, the HAstV8 VP90 sequence was cloned into a pET28a vector, along with a range of truncations at the N-terminus, which are mostly positively charged and unstructured (i.e., aa14–782, aa28–782, aa41–782, and aa72–782) (Fig. 1b). The goal of these truncations was to enhance protein expression level, as the expression of the full-length HAstV8 VP90 could not be detected in the *E. coli* expression system. The expression levels of the four truncation mutants increased as more sequences were removed from the N-terminus (Fig. S1a). Size-exclusion chromatography profiles indicated that VP90^14-782^, VP90^28-782^, and VP90^42-782^ formed exclusively oligomers, while VP90^72-782^ produced mostly dimers (Fig. 2a; Fig. S1b). Considering that VP90^28-782^ was the longest construct that provided sufficient protein yield while retaining an ability to form large protein assemblies, our subsequent studies primarily focused on this protein, as described below.

After purification, the VP90^28-782^ protein was analyzed by a negative-stain EM (Fig. 2b). While the protein maintained its ability to oligomerize, rod-like assemblies instead of icosahedral capsids were observed (27). The assemblies have a regular diameter of ~20 nm with variable lengths. It is hypothesized that this assembly was a result of the heterologous expression system. Previous studies have shown that the acidic domain (AD) orients the C-terminal region of the HAstV VP90 to cellular membranes (29). Without mammalian membranes present during expression, the protein may form non-icosahedral assemblies. While structural and biophysical studies of this rod-like oligomer are ongoing, purified protein samples were utilized for *in vitro* functional assays, as described below.

### Identification of lipid disruption by *in vitro* liposome assay

**Fig 3:**
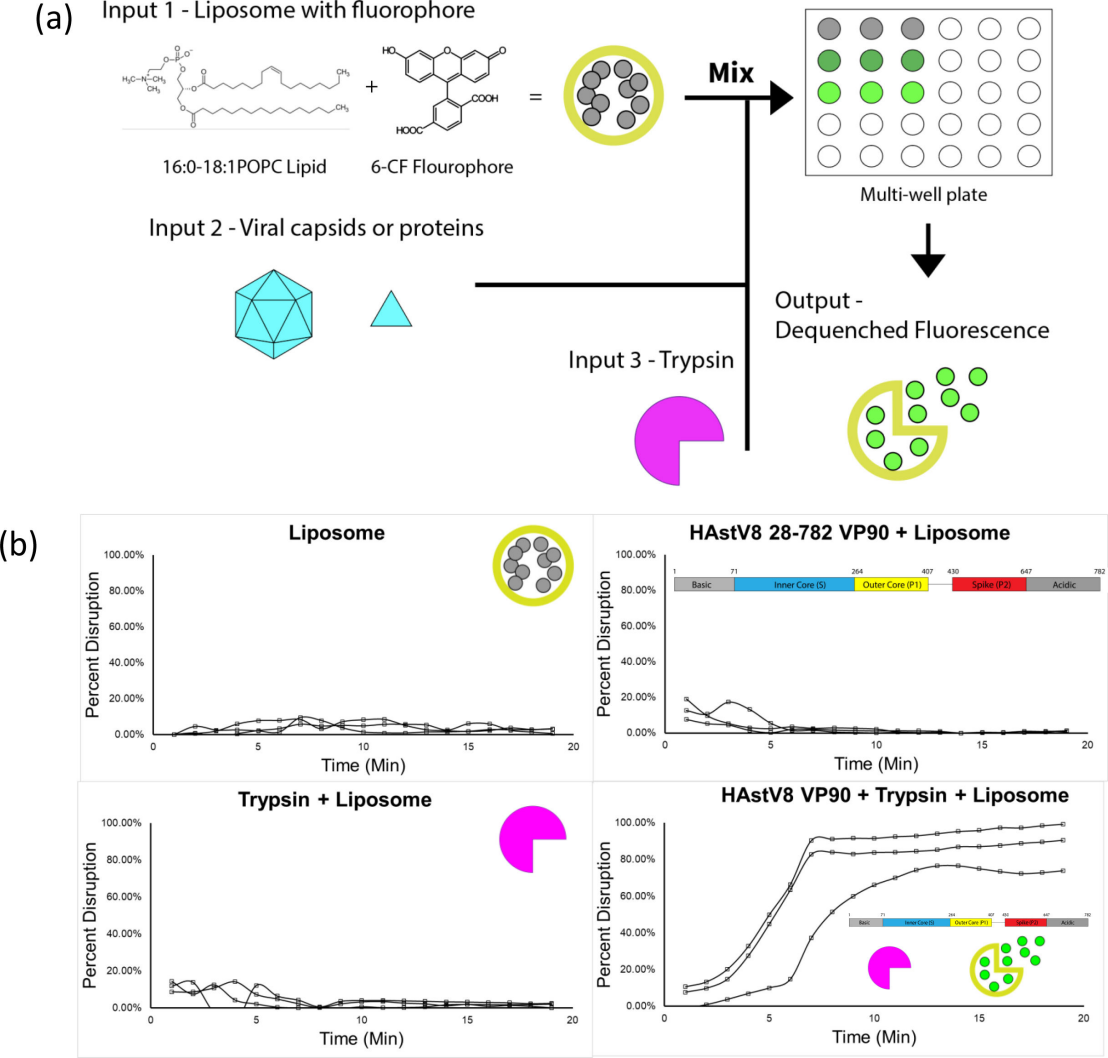
Liposome disruption by HAstV8 VP90. (**a**) Schematic diagram of the lipid disruption assay employed in this study. (**b**) HAstV8 VP90^28-782^ was able to disrupt liposome only in the presence of trypsin. Liposomes were measured to collect a baseline signal for 0% disruption by averaging values at each timepoint (*t* = −5–0 min, not shown), then mixed with trypsin and HAstV8 proteins. The change in fluorescence signal was measured for 20 min in 30-s kinetic intervals (*t* = 0–20 min). All assays were concluded with the addition of 1% Triton X-100 detergent solution to determine the 100% dequenched signal by averaging the values at each timepoint (*t* = 20–30 min, not shown). Each test includes control conditions of liposome alone (top left), HAstV8 VP90^28-782^ mixed with liposomes (top right), trypsin mixed with liposomes (bottom left), and the test condition of HAstV8 VP90^28-782^ mixed with both trypsin and liposomes (bottom right). *N* = 3 technical replicates, with each replicate shown as a separate curve.

With the VP90^28-782^ protein available, the functional properties of the HAstV8 structural polyprotein can be characterized *in vitro*. One important question is how HAstVs acquire the ability to penetrate the host cellular membrane and whether this ability is associated with protease-mediated maturation. Cell culture and animal model-based assays have many confounding factors, making it difficult to assess the exact role of extracellular trypsin in HAstV infection. Hence, we devised an *in vitro* lipid disruption assay to control three main variables: the viral capsids/proteins, the lipid environment, and the protease. For this assay, liposomes containing 1-palmitoyl-2-oleoyl-glycero-3-phosphocholine (16:0-18:1 POPC, or POPC), or other lipid mixes when specified, were generated by extrusion in the presence of self-quenching amounts of the fluorescent dye 6-carbofluorescein (6-CF) and isolated from free dye by size exclusion chromatography (30). This process generates relatively uniform-sized liposomes with quenched 6-CF fluorescence inside the intact liposome. If the liposome bilayer is disrupted, 6-CF leaks out, producing strong fluorescence signal upon dequenching. Protein-mediated lipid disruption activity was measured by the addition of a candidate disruption molecule, either HAstV8 VP90 or individual domains, together with trypsin, which is the protease linked with the increase in HAstV8 infectivity in cell culture (11, 12). These components were added at defined ratios or concentrations to a multi-well plate, and 6-CF-derived fluorescence was measured over time (Fig. 3a).

HAstV8 VP90^28-782^ was the first candidate molecule tested for lipid disruption, as it contains all the structural domains in a multimeric state. Purified HAstV8 VP90^28-782^ and trypsin were mixed with liposomes at time zero, then kinetic measurements were taken over time for 20 min (Fig. 3b). Control experiments included the following: (1) liposomes alone to ensure lipid stability, (2) liposomes mixed with only viral protein, and (3) liposomes mixed with only trypsin. Our results showed that 6-CF release occurred only when both VP90^28-782^ and trypsin were present, indicating that liposome disruption is mediated by a factor activated from VP90^28-782^ upon trypsin treatment, and this activity is independent of any host cellular factors. Trypsin-induced liposome disruption occurred rapidly, showing signs of disruption within minutes of trypsin addition, and approaching maximal intensity within ~10–20 min of viral protein-protease addition (Fig. 3b). Interestingly, pre-treating VP90^28-782^ with trypsin prior to the addition of liposome showed no activity (Fig. S2), indicating a relationship between protein treatment with trypsin and the lipid disruption activity.

### Identification of HAstV8 P1 (aa264–415) as the active domain

**Fig 4:**
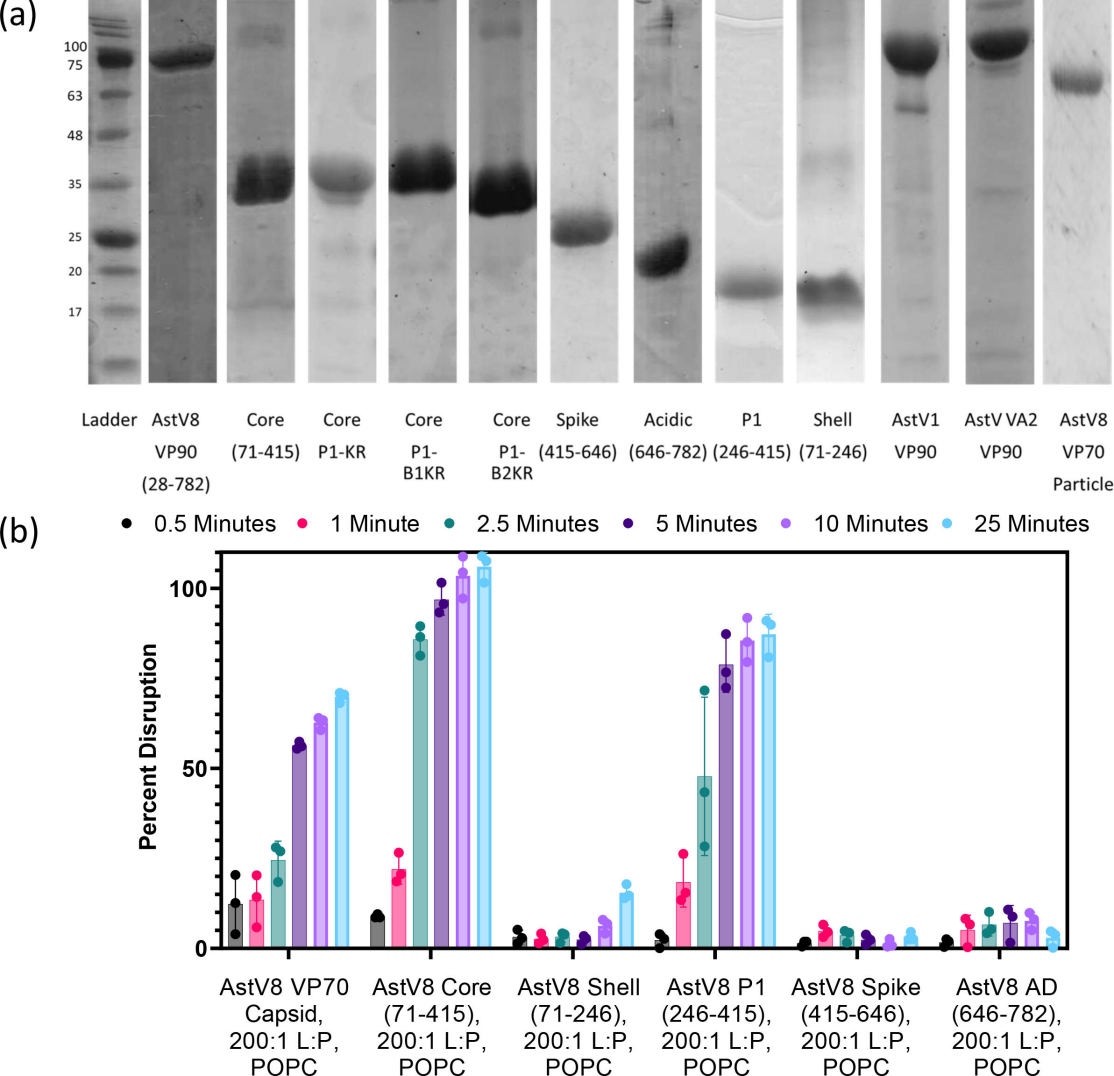
Liposome disruption activity of HAstV8 VP90 mapped to the P1 domain. (**a**) SDS-PAGE gels of purified HAstV8 VP90 domain constructs, purified VP70 particles, and other purified HAstV samples tested for lipid disruption with POPC liposomes. Protein samples were evaluated on separate SDS-PAGE gels and aligned based on the position of a standard protein ladder. All samples were visualized by Coomassie staining. (**b**) Liposome disruption assays. All samples were evaluated at a 200:1 lipid to protein (**L:P**) molar ratio. Fluorescence dequenching was measured in the same kinetic protocol as in Fig. 3b, with percentage disruption at timepoints of 0.5, 1, 2.5, 5, 10, and 25 min shown for clarity. Three technical replicates were performed.

With a lipid disruption activity discovered for AstV8 VP90^28-782^, our next objective was to determine the protein regions necessary and sufficient for this activity. It is possible that the lipid disruption activity is contained in a single domain and can occur independently of a high-order capsid assembly. To test this, each of the VP90 domains, including the core, S shell domain, the P1 domain, the P2 spike domain, and the C-terminal AD, was separately expressed and purified from *E. coli* (Fig. 1b; Fig. 4a). Considering that VP90^28-782^ forms a rod-shaped structure that is different from the icosahedral capsid of a native virus, we also tested whether the same membrane disruption activity is possessed by authentic VP70 particles, which were purified from Caco-2 cells infected with HAstV8 with trypsin inhibitors. VP70 and sub-domain constructs were mixed with trypsin and liposomes at the consistent molar ratios (1:100 trypsin:protein, 1:200 viral protein:lipid) and the resulting kinetic timepoints were recorded from 0.5 to 25 min. As expected, VP70 immature particles exhibited membrane disruption activity when incubated with trypsin and fluorophore-filled liposome (Fig. 4b). In addition, both core (71–415) and P1 (246–415) demonstrated similar levels of activity and kinetics, while the shell, spike, and AD domains were completely inactive. Therefore, our results support the hypothesis that the P1 domain is both necessary and sufficient for lipid disruption after trypsin treatment. The data also indicate that the assembly context is not necessary for the lipid disruption activity, as the core and P1 domains stayed as monomers in solution.

### Trypsin cleavage in the HAstV8 P1 domain is necessary for lipid disruption

**Fig 5:**
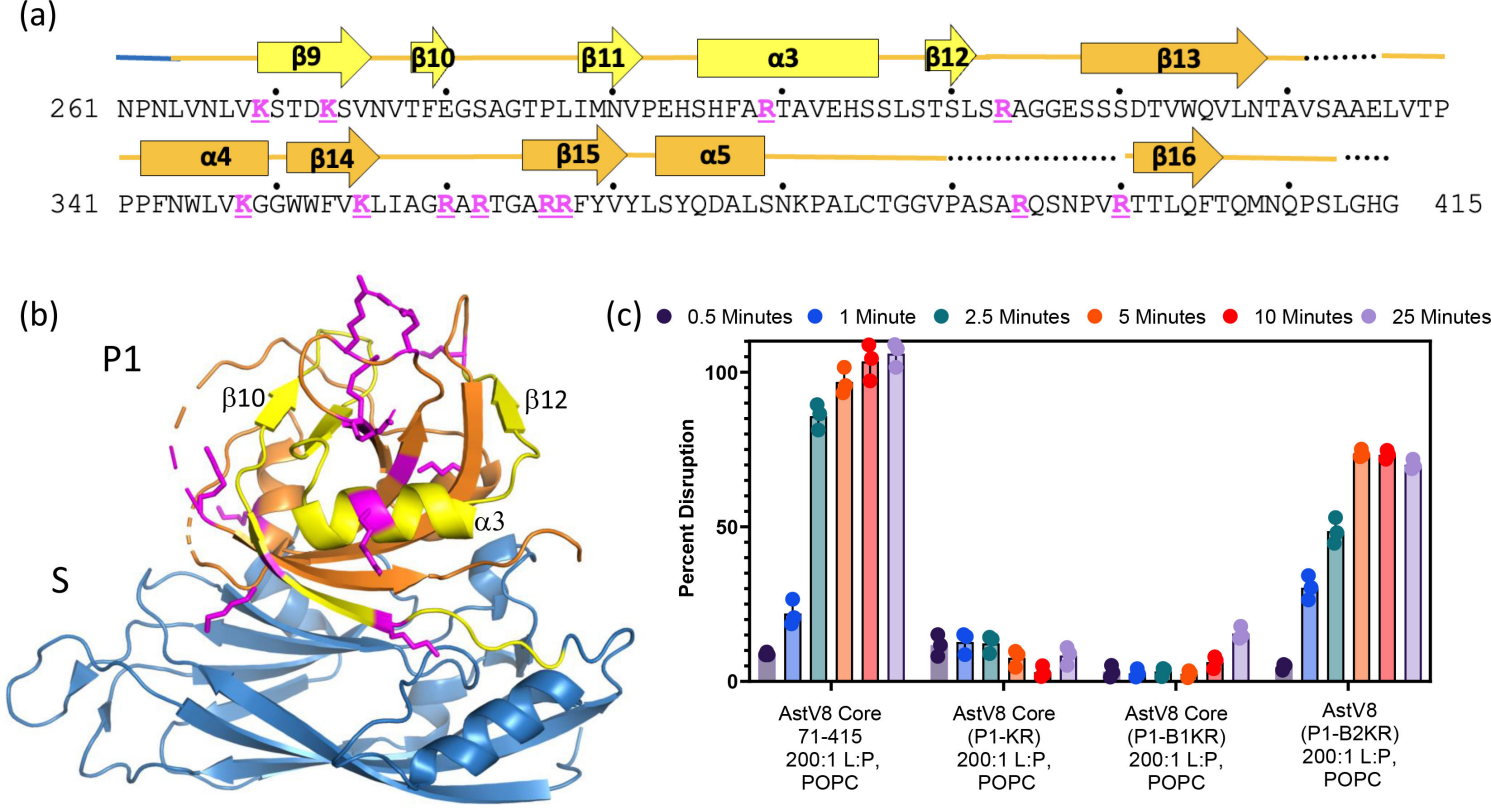
Trypsin cleavage sites from amino acids 264-313 of the HAstV8 Core are essential for liposome disruption. (**a**) Amino acid sequence of P1, with β-sheets labeled with arrows and α-helices with blocks. Trypsin cleavage sites are highlighted in magenta, with four sites from aa264 to 313 (yellow) and eight sites from aa314 to 415 (orange). (**b**) The structure of the HAstV8 core with the 12 trypsin cleavage sites mapped to P1. The shell domain is in blue and P1 domain is in orange (block-1 region)/yellow (block-2 region). Lysine and arginine residues are highlighted by magenta sticks. (**c**) Liposome disruption by P1 mutants. Trypsin cleavage site removal in Core(P1-KR) and Core(P1-B1KR) resulted in the loss of liposome disruption activity. All mutant proteins were mixed with liposomes at the same 200:1 lipid-to-protein molar ratio.

With the candidate lipid disruption factor localized to the P1 domain, we hypothesize that trypsin treatment of P1 releases a peptide with membrane disruption activity. Protein sequence analysis of the P1 domain shows 12 trypsin cleavage sites, including four lysine residues and eight arginine residues (Fig. 5a). K380 is excluded from this list because it is followed by a proline which makes this site unsuitable for trypsin cleavage. Based on their distribution on the primary sequence, these 12 sites can be clustered into two blocks: block-1 containing four basic residues (K269, K273, R299, and R313) and block-2 with eight basic residues (K347, K354, R359, R361, R365, R366, R393, and R399), that are separated by a 33-aa long region free of any trypsin site. Of 12 trypsin cleavage sites, 10 sites are mapped to the surface of the P1 domain except for K269 and K354 which are partially buried (Fig. 5b).

To determine which trypsin cleavage sites are important for generating the membrane lytic peptide, these lysine and arginine residues were mutated to glutamines, yielding three mutants, including Core(P1-KR) (all 12 sites mutated), Core(P1-B1KR) (four sites in block-1 mutated), and Core(P1-B2KR) (eight sites in block-2 mutated). Glutamine mutations were chosen over alanine and serine because the latter often resulted in insoluble proteins and low protein yields likely due to protein folding issues.

When mutant proteins were used in liposome disruption assays, our results showed that Core(P1-KR), which has all 12 trypsin cleavage sites removed in the P1 domain, completely lost the membrane disruption activity (Fig. 5c). The two block mutants exhibited different behaviors: while Core(P1-B1KR) was inactive, Core(P1-B2KR) maintained >50% of the lipid disruption capacity when compared to the WT core. Therefore, these two block mutants allowed us to attribute the membrane disruption activity to the P1 region ranging from amino acid residues 264–313. The Core(P1-B2KR) construct had reduced activity in terms of percent disruption at completion of the assay compared to the WT core, possibly due to differences in structural conformation and/or the absence of peptides from the P1 block-2 region that may play an auxiliary role in membrane disruption.

### Identification of lipid-associated products by mass spectroscopy

**Fig 6:**
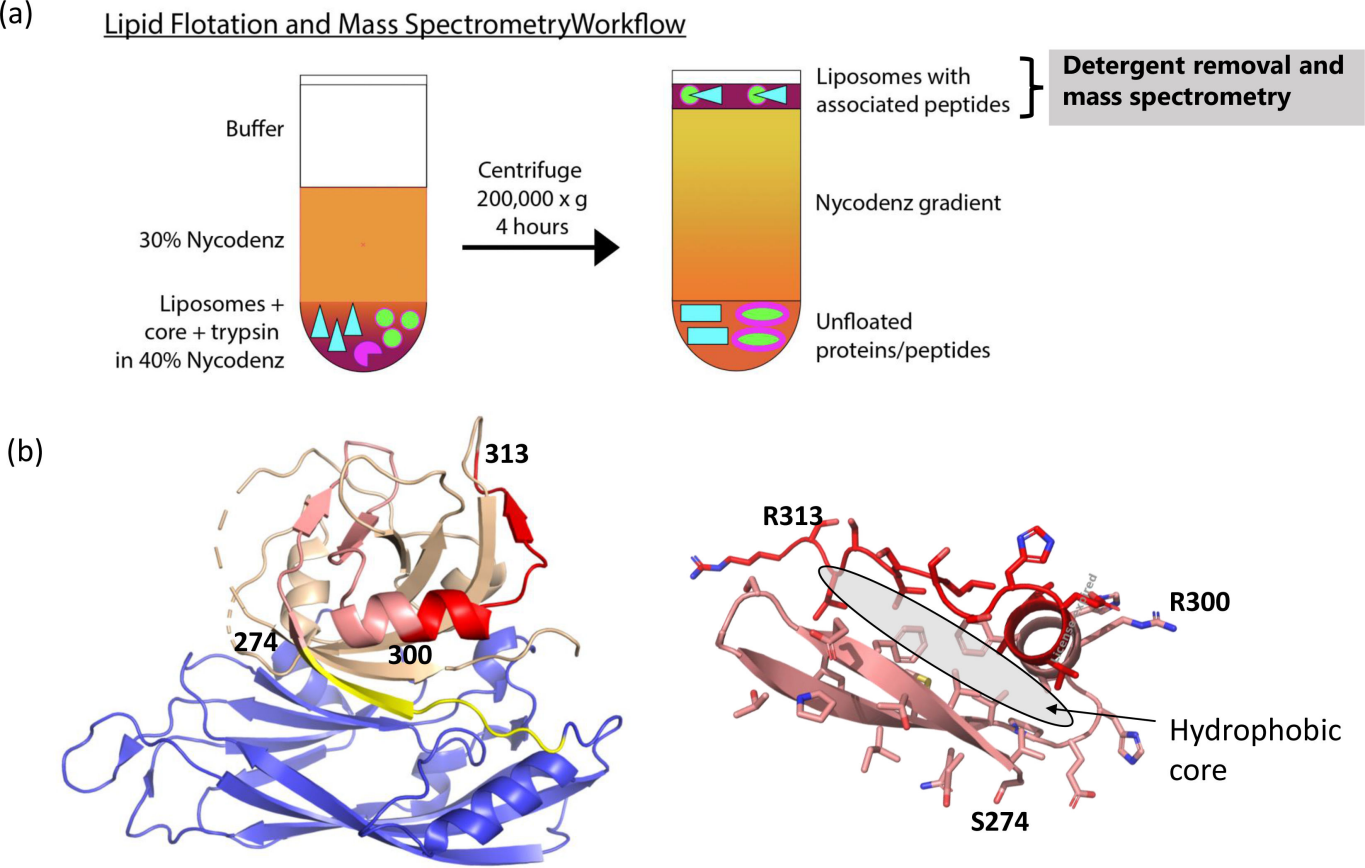
Mass spectroscopy identification of HAstV8 liposome-associated peptides. (**a**) A schematic of the protein-associated liposome flotation assay. (**b**) Structural modeling of the liposome-associated peptides. (Left) Crystal structure of the core with aa300–313 highlighted in red and aa274–299 in pink. The shell domain is shown in blue, and the rest of the P1 region is colored in yellow and orange as in Fig. 5a. (Right) The predicted structure of aa274–313 with a hydrophobic core that could be exposed upon liposome insertion.

Given the high efficiency of the lipid disruption activity, it is very likely that a peptide generated by trypsin maturation is directly integrated into the lipid environment, in the form of an amphipathic peptide or another type of sequence that is capable of membrane pore formation (23, 31). Transmission electron microscopy (TEM) images of disrupted liposomes were structurally similar to those of intact ones, indicating that the overall morphology of the liposomes was largely preserved and not solubilized by the lytic peptide (Fig. S3). To identify the liposome-associated peptide from HAstV8, we isolated liposomes from liposome disruption assays by flotation in a density gradient (Fig. 6a). In brief, HAstV8 core protein, trypsin, and liposomes were incubated for 5 min, and the reaction mixture was mixed with Nycodenz to a final concentration of 40%. This mixture was deposited at the bottom of an ultracentrifuge tube, which was then layered with 30% Nycodenz and buffer. After high-speed centrifugation, the floated material on top of the gradient was collected, proteins and peptides were concentrated by trichloroacetic acid (TCA) precipitation, and the resulting protein products were determined by mass spectroscopy.

Our mass spectroscopy data showed that the top hit among liposome-associated peptides is aa300–313 (Table 1). The second hit was peptide aa274–299, which immediately precedes aa300–313 in primary sequence. Other peptide hits include aa180–188, aa115–136, and aa179–188 from the shell domain. These minor shell-derived peptides likely had non-specific interactions with the lipid, as the shell domain itself is incapable of liposome disruption (Fig. 4). In the context of the core structure, aa274–313 forms an extended structural element that is partially buried, comprised of a β-hairpin followed by an α-helix and a short β-strand (Fig. 6b). Alphafold2 prediction of the peptide aa274–313 on its own yielded a more compact structure with a β-hairpin stacked against an α-helix (Fig. 6c) (32). This structure is stabilized by multiple hydrophobic residues at the interface between the β-hairpin and the α-helix. It is conceivable that the peptide aa274–313 may adopt an alternative conformation different from the Alphafold2 model in the lipid environment, and those hydrophobic residues may facilitate the insertion of the peptide into the lipid bilayer.

**TABLE 1:** Liposome-associated peptides from HAstV8 core 71–415 identified by mass spectrometry^*a*^

Peptide sequence	Relative confidence	PSM#	Modification	Amino acid range in VP90
TAVEHSSLSTSLSR	High	76		300–313
SVDVTFEGSAGTPLImNVPEHSHFAR	High	1	M16(Oxidation)	274–299
SVDVTFEGSAGTPLIMNVPEHSHFAR	High	3		274–299
HLDVTVGK	High	3		180–188
DATGSTQFGPVQALGSQYSMWK	High	4		115–136
KHLDVTVGK	High	1		179–188
tAVEHSSLSTSLSR	High	1	N-Term(Acetyl)	300–313

^*a*^ PSM# represents the number of identified peptides of that species.

### Lipid disruption function is maintained in other HAstV strains and lipid identities

**Fig 7:**
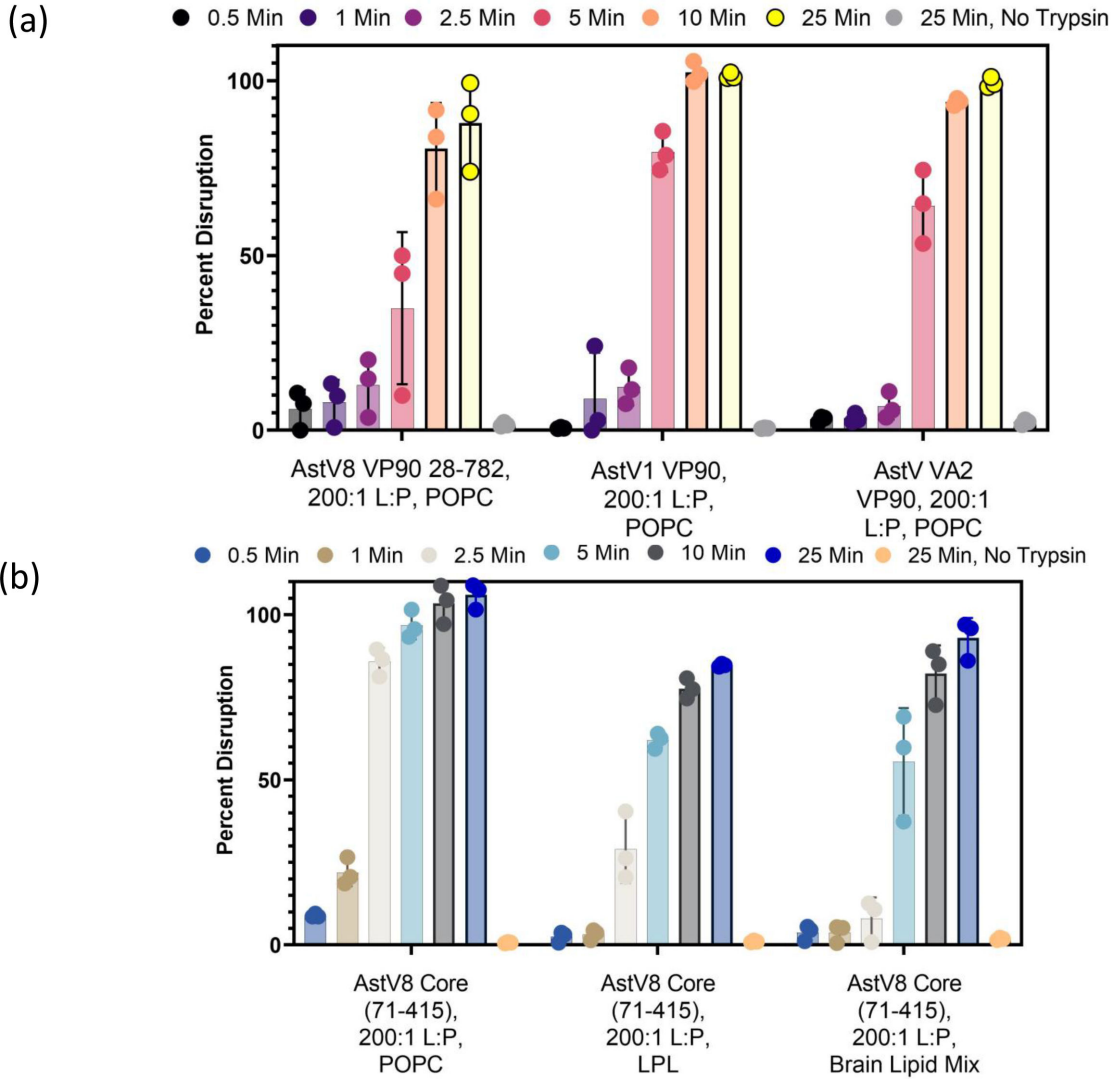
Liposome disruption activity of HAstV capsid proteins observed using different virus strains and different lipids. (**a**) Liposome disruption by HAstV1 and VA2 VP90. The L:P ratio and trypsin concentration were maintained from the HAstV8 test conditions. (**b**) Liposome disruption of HAstV8 core tested in different lipids.

Considering that all classical HAstVs of the eight serotypes require trypsin for maturation and infectivity, it is possible that the capsid proteins of all eight HAstV serotypes possess a similar liposome disruption activity. The structure and the maturation process of the HAstV1 capsid have been extensively characterized, so it is a prime candidate to test whether this function is conserved across HAstV classical strains (9, 10). In addition to HAstV1, the capsid protein of the non-canonical HAstV VA2 strain from the VA clade was tested. HAstV VA2 capsid protein only shares a ~30% amino acid sequence identity with HAstV8. Although HAstVs in the VA clade do not require trypsin for propagation *in vitro* (33), it was recently reported that HAstV VA1 is proteolytically matured by an intracellular protease (34). HAstV1 and VA2 VP90 baculovirus production vectors were readily available from Dr. Stacey Schultz-Cherry, which were utilized for protein expression and purification from insect cells (Fig. 4a). When tested, the VP90 precursor proteins of both HAstV1 and VA2 strains were capable of liposome disruption when treated with trypsin (Fig. 7a). These results strongly indicate that the HAstV lipid disruption function is preserved across both classical and VA HAstV strains, even though different host proteases may be used for the maturation of these viruses during natural infection.

Biological membranes contain a variety of lipids and the composition may vary in different cell types and even at different regions of the same cell. Therefore, it is important to explore how lipid composition, which ultimately determines fluidity, charge, and local stability of the membrane, affects the HAstV lipid disruption activity. Liposomes made with 100% POPC provide a model lipid environment but lack many of the properties of cellular plasma membrane, such as a heterogeneous lipid environment, charged lipids, and other commonly found lipids such as cholesterol. To test this, liposomes were made from two different cellular lipids, a liver polar lipid (LPL) mix (Avanti cat. no. 181108) that contains a large variety of lipid types, and a brain total lipid extract (Avanti cat. no. 131101) that has a largely undefined lipid composition. Our results showed that the HAstV8 core exhibited similar levels of liposome disruption activity in cell-derived lipids compared to POPC. The slight reduction in the rate and capacity of disruption in the cell-derived lipids may be due to constraints from certain components of these lipid mixes or slightly different modes of interactions between capsid-derived peptides and cellular lipids.

## DISCUSSION

This work presents the first *in vitro* evidence that HAstV virions can disrupt host cellular membranes in the presence of trypsin to facilitate viral entry. The dependence of HAstV maturation by host proteases including trypsin has been established previously *in vivo* and *in vitro* (35). Upon trypsin treatment, immature HAstV8 VP70 particles are converted to mature capsids consisting of three major polypeptide species: VP34, VP27, and VP25 (Fig. 1). Large-scale structural rearrangements occur after trypsin-mediated maturation, with immature VP70 showing 90 capsid spikes and mature VP34/VP27/VP25 particles showing only 30 spikes. This maturation process is able to increase the virus infectivity by 100–1000 fold, but the mechanistic basis of the enhanced infectivity is not well understood (11, 12). Our observation of a membrane disruption activity by the HAstV8 capsid precursor protein VP90 that is dependent on trypsin treatment strongly suggests that membrane penetration is the key for infectivity. Trypsin treatment of immature VP70 capsids likely releases one or more peptides capable of membrane penetration.

Systematic subdomain testing of the HAstV8 VP90 revealed that the lipid disruption function resides in the P1 domain (aa264–415), and the P1 domain alone is sufficient for the lipid disruption activity (Fig. 4). Site-directed mutagenesis showed that mutating four trypsin cleavage sites altogether (K269, K273, R299, and R313) in the P1 domain resulted in the complete loss of activity. Mass spectrometry of floated liposomes preincubated with HAstV8 core and trypsin identified several lipid-binding peptides, with aa300–313 and aa274–299 being the two top hits. Because peptides/proteins extracted from floated liposomes were extensively treated with trypsin prior to peptide identification by LC/MS/MS, it is possible that these two peptides were digested products from a longer precursor with R313 at the C-terminus. It is interesting to note that VP34 found in mature HAstV8 particles likely terminates at R313 (16, 17). Therefore, trypsin treatment may be necessary for HAstV8 infectivity as it allows a lytic peptide to become liberated at the residue R313, in a manner similar to membrane-disrupting polypeptides generated in other non-enveloped viral entry processes (24).

Of the three predicted trypsin cleavage sites preceding R313 in the P1 region of VP34, K269 is partially buried, K273 is part of a β-sheet, and R299 is in the middle of an α-helix. Considering their partially buried nature and structural rigidity, none of these three predicted sites appears to be highly susceptible to trypsin cleavage. Therefore, VP34 can remain stable in the context of an icosahedral viral capsid when treated with a limited amount of trypsin. Once encounters a suitable environment, VP34 likely mediates membrane penetration of HAstV8 by inserting its C-terminus into the host membrane. Because the VP34/27/25 particles are stable in solution, certain stimuli may be needed to trigger this insertion reaction under physiological conditions, such as membrane proximity, acidic pH, protein unfolding, thorough digestion by host proteases, etc. The predicted structure of the peptide aa274–313 consists of a β-hairpin packed against an α-helix with an extensive hydrophobic core (Fig. 6). Residues at the hydrophobic core may facilitate the insertion of the peptide into the membrane when the peptide adopts an extended conformation.

Our results showed that pretreating the HAstV8 VP90 with trypsin before mixing with lipids led to the loss of liposome disruption activity. This may be due to over-digestion of VP90, resulting in the release of small lytic peptide(s) that are unstable in the absence of lipids (Fig. S4), in contrast to mature HAstV8 virus where VP34 is stable in the context of an icosahedral capsid. Alternatively, this may imply that the heterologous expression of VP90^28-782^ produces a different tertiary/ternary structure from authentic VP70 particles, such that the trypsin-induced factor soon loses its activity in the absence of lipids. It is also possible that other viral/cellular factors from natural infection may help to preserve the trypsin-induced factor in an active conformation during cellular uptake or endosomal degradation to ensure proper host entry.

In addition to HAstV8, we found that the VA2 and HAstV1 VP90 also possess the lipid disruption activity upon trypsin treatment, suggesting that this activity may be conserved in other HAstVs. Multi-sequence alignment shows that the residue R313 is strictly conserved in all eight serotypes of classical HAstVs (Supplemental Information; Fig. S5). In VA2 and MLB1 strains, the sequence between aa274 and 313 is poorly conserved, which is not surprising considering that HAstVs in the VA clade are likely matured by a host intracellular protease (Supplemental Information; Fig. S6). Future work is needed to solve the structure and to map the C-terminal residue of the HAstV8 VP34-equivalent fragment in VA2, MLB1, and other HAstVs. Site-directed mutagenesis will also help to confirm the importance of the cleavage site in membrane penetration using *in vivo* infection assays or *in vitro* liposome disruption tests.

Our finding that HAstV8 VP90 can be isolated in large quantities from *E. coli* allows us to study HAstV assembly and maturation mediated by host proteases (caspases, trypsin, etc.) *in vitro*. The observation of rod-shaped protein assemblies not seen in cell-culture derived virions supports earlier hypotheses that eukaryotic host membranes and/or viral nucleic acids are required for proper virus assembly (29). The liposome disruption assay used in this study provides a simple system for a thorough investigation of the membrane penetration activity mediated by HAstV capsids. While more complex models such as human cell culture or established AstV animal infection models may provide a more authentic biological context, the *in vitro* liposome assay avoids complex factors such as extracellular proteases, host innate immunity, and dynamic lipid environments. Future studies will benefit from using this *in vitro* system to assay a large range of virus samples, candidate peptides, lipids, proteases, or other environmental conditions to establish the mechanistic basis of capsid-mediated lipid disruption in HAstV infection and pathogenesis.

## MATERIALS AND METHODS

### Molecular cloning for *E. coli* expression

The VP90 sequence coding for HAstV Yuc8 (HAstV8) VP90 (Genbank # AF260508.1) and its N-terminal truncations were cloned into the multiple cloning site of the pET-SUMOc vector with an N-terminal 6xHis-SUMO tag. Full core (aa71–415), shell (aa71–264), spike (aa415–646), and AD (aa646–782) constructs were cloned into a pET28a vector. The P1 domain (aa264–415), P1-KR, P1-B1KR, and P1-B2KR constructs were cloned into a pET32 vector.

Trypsin site mutations in the core construct were made by ordering GeneBlocks (IDT) containing the desired mutations, which were then inserted into the core backbone using Gibson assembly. These mutations include block mutants changing arginine and lysine codons to alanine, serine, or glutamine codons for all sites between amino acids 264–415, 264–315, and 315–415.

All construct sequences were confirmed by sequencing using T7 primers.

### *E. coli* expression and purification

Rosetta 2 (DE3) *E. coli* or BL21 were transformed with expression vectors and grown on lysogenia broth (LB) plates with antibiotic selection (Day 0). A colony was used to inoculate 5 mL of LB and grown overnight. The overnight culture was used to inoculate 500 mL of teriffic broth (TB) with 1.5% (volume/volume) ethanol and grown to 1.4–1.6 optical density (OD). This culture was then induced with a final concentration of 1 mM isopropyl β-d-1-thiogalactopyranoside (IPTG) and incubated for 18–20 h at 16°C. Cells were collected and lysed using a sonicator. The lysis buffer used during sonication contained 50 mM Tris pH 8.0, 300 mM NaCl, 1 mM NaN3, 20 mM imidazole, 0.1% NP-40, 1 mM ethylenediaminetetraacetic acid (EDTA), 0.1 mg/mL of lysozyme, and a cocktail of protease inhibitors including PMSF, leupeptin, and pepstatin.

After lysis, the soluble lysate was separated from insoluble fractions by centrifugation at 15,000 g for 1 h. The supernatant was applied to a 5-mL HisTrap Column (Cytiva), which was then washed with lysis buffer and eluted with a gradient of increasing imidazole up to a final concentration of 1 M. Further purification was conducted on a Superdex-200 or Superdex-75 size exclusion column using an elution buffer containing 50 mM Tris-HCl pH 8.0, 150 mM NaCl, and 1 mM NaN3. Fractions containing the target protein were collected, concentrated, and analyzed by sodium dodecyl sulfate-polyacrylamide gel electrophoresis (SDS-PAGE) and Western blot. Protein concentrations were determined based on A280 UV absorption and calculated extinction coefficients.

### Insect cell expression and purification

The pFastbac plasmid constructs for HAstV1 and VA2 capsid precursor protein with a C-terminal 6xHis tag were provided by Dr. Schultz-Cherry at St. Jude Children’s Research Hospital. The plasmids were transformed into DH10Bac *E. coli* competent cells to produce recombinant bacmids. These were used to generate the P1 and P2 generation of baculoviruses. Five hundred milliliters of Sf21 cell at a density of 1 × 10^6^ cells/mL cells was infected with recombinant baculovirus at a multiplicity of infection (MOI) of 5 and collected at 72-h post-infection. Insect cell pellets were processed using the same reagents and the same protocol as for *E. coli* cells described above.

### Virus growth and purification

HAstV8 virions were prepared as previously described (36). Confluent monolayers of Caco-2 cells were infected with HAstV-8, strain Yuc8, previously treated with 200 µg/mL of trypsin (Gibco, 27250018) for 30 min at 37°C. After this time, trypsin was inactivated with 200 µg/mL of soybean trypsin inhibitor (Sigma, T9003), and the virus was then added to cell monolayers at an MOI of 1, and adsorbed for 1 h at 37°C. The inoculum was removed, and the cells were washed twice with Minimal Essential medium (MEM). Then, Advanced MEM (Gibco 12492013) containing 5% fetal calf serum was added and the cells were incubated for 18 h at 37°C. Then, the cells were frozen and thawed three times, and the resulting cell lysate was subsequently clarified by centrifugation at 2,000 × g for 10 min at 4°C. The supernatant was filtered through a 0.45-µm nitrocellulose membrane, and the clarified and filtered cell lysate was ultracentrifuged at 50,000 × g for 16 h at 4°C. Finally, the pellet was resuspended in 4 mL of 0.5% β-octylglucoside in TNE buffer (50 mM Tris-HCl, pH 7.4, 0.1M NaCl, 10 mM EDTA) and incubated for 30 min at 4°C to release the virus particles that could be associated with cellular membranes. This sample was then centrifuged through a 1 mL of 30% sucrose cushion in TNE buffer, at 200,000 × g for 2 h at 4°C. The pellet was resuspended in 500 µL of TNE and stored at −70°C until use. The concentration of the virus was determined by Coomassie blue staining of a sample analyzed by SDS-PAGE, using as reference a bovine serum albumin (BSA) concentration standard curve.

### Negative staining TEM

To prepare negative staining grids, FCF400-Cu grids (Electron Microscopy Sciences) were pretreated by glow discharge at 5 mA for 1 min. Three microliters of the protein solution was added onto the grid and left to absorb for 1 min. The protein solution was removed from the grids by blotting with Whatman paper. The grids were then rinsed once with distilled water and stained with freshly prepared 0.75% uranyl formate solution for 30–90 s. After air-drying overnight, the grids were examined using a JEOL 1230 High Contrast TEM at 80 kV. Images were recorded on a Gatan CCD detector and analyzed using ImageJ.

### Liposome preparation

Liposomes were prepared as previously described (30). In short, lipid stocks were resuspended in chloroform (10 mg/mL stock lipid concentration). Along with the base lipid stocks, Rhodamine-labeled POPC was added at a 1:100 molar concentration. The lipid mixes were dried under a stream of N2 gas forv~10 min, then transferred to a desiccator for 45 min. The lipid was resuspended by the addition of a buffer consisting of 150 mM NaCl, 50 mM Tris, 1 mM NaN3, and 20 mM 6-CF and vortexing for 15 min at room temperature. The hydrated lipids were then put through 10 freeze–thaw cycles in liquid nitrogen to minimize multilamellar liposomes. To form the liposomes at the correct size, an Avanti mini extruder was fitted with 100 nm pore size polycarbonate filters and passed through alternating syringes 19 times. The extruded liposomes were run through an equilibrated PD Spin-Trap G25 (Cytiva, Cat no. 28918004) to remove unencapsulated 6-CF.

To determine total lipid concentration of liposomes, a sample of crude lipid at 10 mg/mL was collected and read on a Tecan Infinite 200 Pro plate reader for rhodamine emission at 590 nm, then compared to a sample of the extruded lipids. The liposomes were stored at 4°C for up to 2 weeks. This protocol was used for any variation of lipid tested, including 100% POPC, a LPL extract (Avanti, Cat no. 181108), and a Brain Extract Polar Mix (Avanti, Cat no. 141101).

### Liposome disruption assay

Lipid disruption activity was measured using a Tecan Infinite 200 Pro. To each reaction well, lipid was added based on rhodamine quantification to a final amount of 25 μg per reaction. Buffer was added to a final volume of 50 μL. The samples were measured for dequenched fluorescence at 512 nm every 30 s in a kinetic assay. Untreated liposomes were measured for 5 min (*t* = −5–0, not shown on graph) and signal values for each measurement timepoint were averaged to establish a baseline for 0% disruption. After baseline observation, trypsin (Worthington Biochemical Corporation, Cat no. LS003740) was added to all the reaction wells to a final amount of 2 μg. At the end of the observation period, a detergent such as 20% Triton X-100 was added to fully disrupt the liposomes. The fully disrupted liposomes were measured for another 10 min (*t* = 20–30, not shown on graph) and signal values for each measurement timepoint were averaged to establish a signal limit for 100% disruption. Three technical repeats were performed for each assay conditions.

### Liposome flotation

Density gradient liposome isolation was adapted from an existing protcol (30). Capsid core protein, extruded lipid material, and trypsin were mixed at a molar ratio of 1:200 capsid protein to lipid with 2 μg trypsin to a final volume of 150 μL. The mixture was incubated at room temperature for 10 min, and then 150 μL of 80% Nycodenz (Progen) stock made in the same buffer was added to make a 40% Nycodenz/reaction solution. This was added to the bottom of a 5 × 41 mm^2^ tube. A 250 μL layer of 30% Nycodenz was applied, followed by a layer of buffer with 0% Nycodenz. The layered tubes were placed in a SW 55Ti rotor and spun at 50,000 rpm for 4 h with no break during slowdown. Each of the resulting layers was collected based on rhodamine signal from the lipids.

### Western blot

Western blot samples were run on an SDS-PAGE gel and transferred to polyvinylidene difluoride membrane. After blocking with 5% milk in Tris-buffered saline (TBS, pH 7.4) containing 0.1% Tween-20 (TBST), membranes were probed with primary antibodies for the 6x HisTag (4E3D10H2/E3, ThermoFisher) overnight at 4°C. Membranes were then washed three times with TBST and incubated with horseradish peroxidase-conjugated secondary antibodies for 1 h at 4°C. After washing three times with TBST, immune-reactive bands were detected using SIGMAFASTTM BCIP/NBT alkaline phosphatase substrate (Sigma).

### Purification and identification of lipid-associated proteins

To remove the lipid from any protein/lipid complex, the top layer from centrifugation was diluted to 1 mL in buffer with a final concentration of 0.2% Triton-X 100 to fully disrupt the lipids. Residual protein was precipitated by the addition of 250 μL of a TCA solution (10 g dissolved in 7 mL distilled H20) and incubation at 4°C for 10 min. The mixture was centrifuged at 4°C for 5 min at 14,000 rpm. The tube was collected, supernatant poured off, and 200 μL of chilled acetone was added to wash the pellet without resuspension. Centrifugation and wash steps were repeated until rhodamine–lipid coloration is removed from the pellet. The pellet was dried at 50°C for 5 min to remove acetone, and 40 μL of buffer was added with vigorous pipetting to resuspend the protein. The suspended protein solution was applied to discontinuous gel electrophoresis, and as the sample migrated to the end of stacking gel, the gel slice containing the dye was cut out and used for proteomic profiling at the Clinical and Translational Proteomics Service Center at the University of Texas Health Science Center at Houston. At the Proteomics Service Center, gel band samples were subjected to in-gel digestion as previously described (37). An aliquot of the tryptic digest (in 2% acetonitrile/0.1% formic acid in water) was analyzed by LC/MS/MS on an Orbitrap Fusion Tribrid mass spectrometer (Thermo Scientific) interfaced with a Dionex UltiMate 3000 Binary RSLCnano System.

## SUPPLEMENTAL MATERIAL

Fig. S1 to S6 jvi.00802-23-s0001.pdf PDF version of Supplemental figures. Higher-resolution version available upon request.

ASM does not own the copyrights to Supplemental Material that may be linked to, or accessed through, an article. The authors have granted ASM a non-exclusive, world-wide license to publish the Supplemental Material files. Please contact the corresponding author directly for reuse.
