# Supplementary material for: Human astrovirus capsid protein releases a membrane lytic peptide upon trypsin maturation
Source: J Virol. 2023 Jul 28;97(8):e00802-23. doi: 10.1128/jvi.00802-23 (PMC10506485; doi:10.1128/jvi.00802-23)
Supplement: Fig. S1 to S6 — PDF version of Supplemental figures. Higher-resolution version available upon request. [file jvi.00802-23-s0001.pdf]

Human astrovirus capsid protein releases a membrane lytic peptide upon trypsin treatment

Supplemental Information

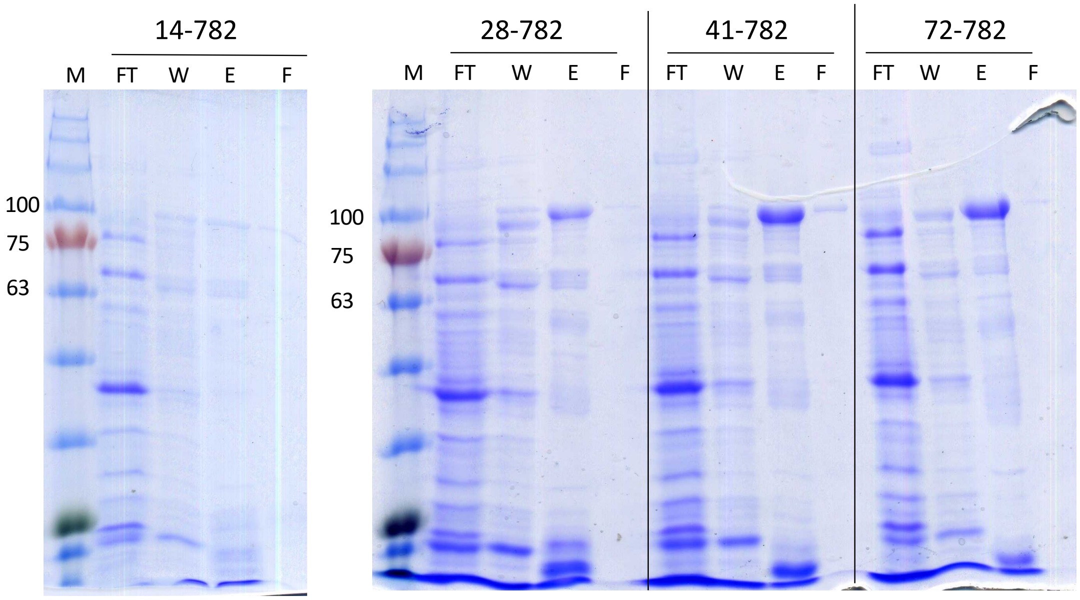

FT: flowthrough; W: 25mM Imidazole; E: 250mM Imidazole; F: 500mM Imidazole

Figure S1a. VP90 constructs purified by Ni-NTA affinity. One liter of cells were used for each construct.

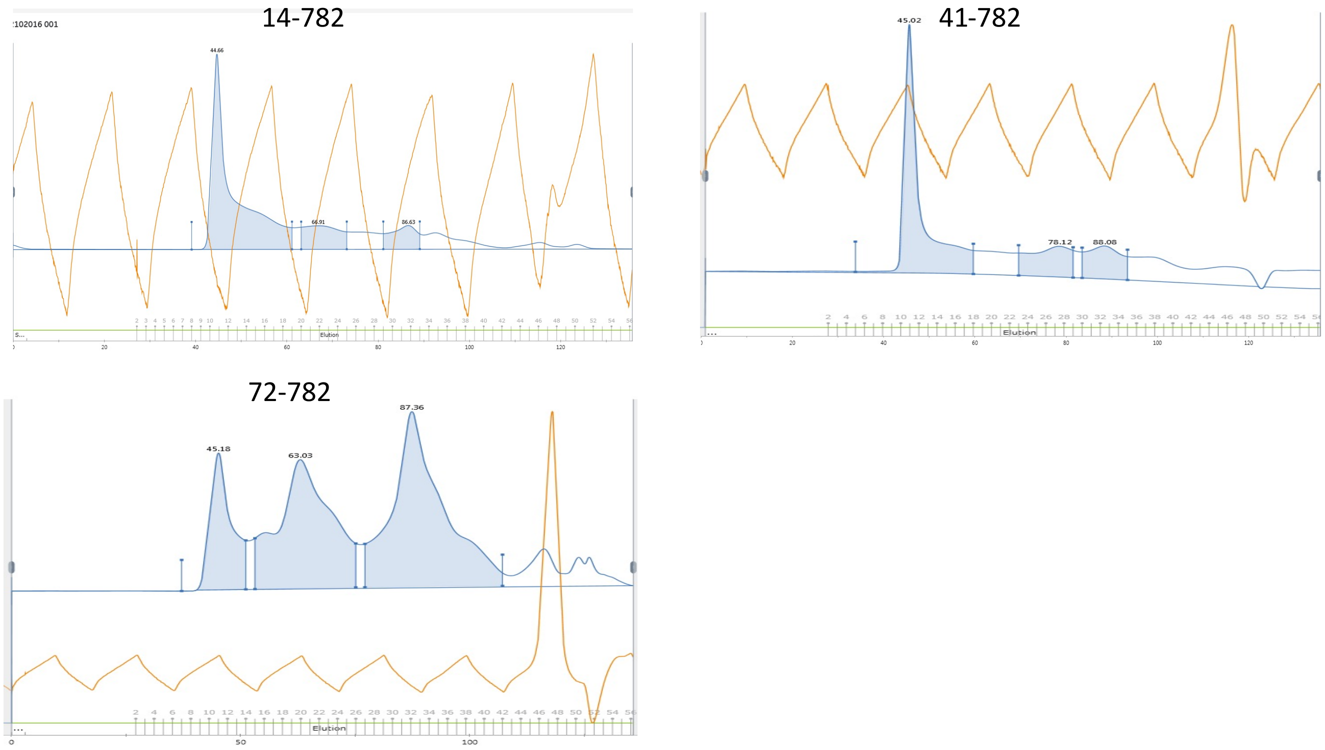

Figure S1b. VP90 constructs purified by gel filtration chromatography using a S-200 column.

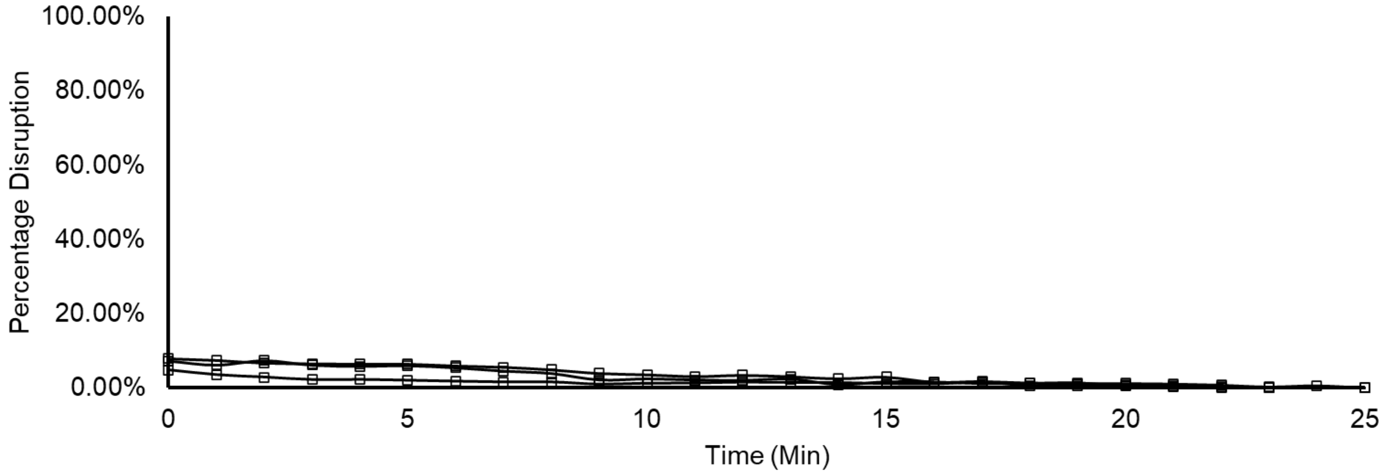

Figure S2. Kinetic trace of the disruption activity from AstV8 VP90 mixed with trypsin before addition to liposomes. Kinetic data was collected in the same manner as other lipid disruption assays, with disruption percentage shown at each 1-minute interval. Trypsin was mixed with VP90 5 minutes prior to liposome addition at t = 0.

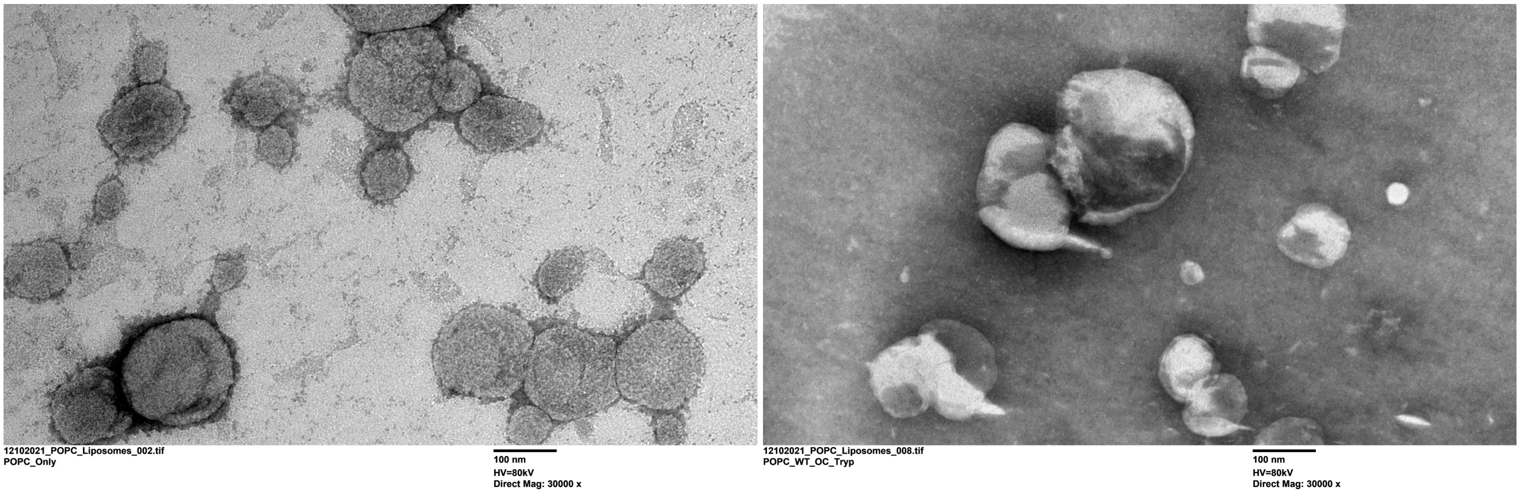

Figure S3. Transmission electron microscopy (TEM) images of POPC liposomes treated with (a) AstV8 71-415 Core protein alone or (b) AstV8 71-415 Core protein and trypsin.

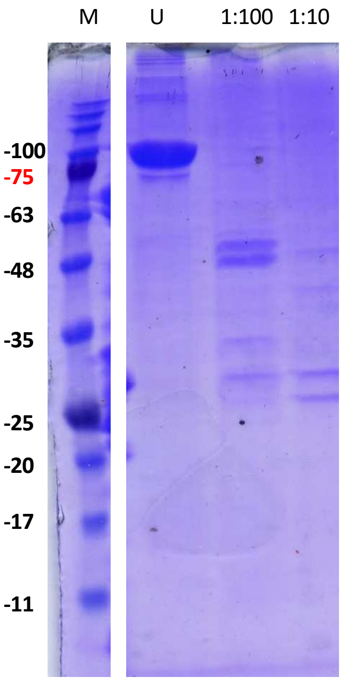

Figure S4. AstV8 $VP90^{28\text{-}782}$ digested with trypsin. All samples were incubated at 37° C for 5 minutes. U = untreated, 1:100 = 1:100 molar ratio of trypsin to $VP90^{28\text{-}782}$, 1:10 = molar ratio of trypsin to $VP90^{28\text{-}782}$

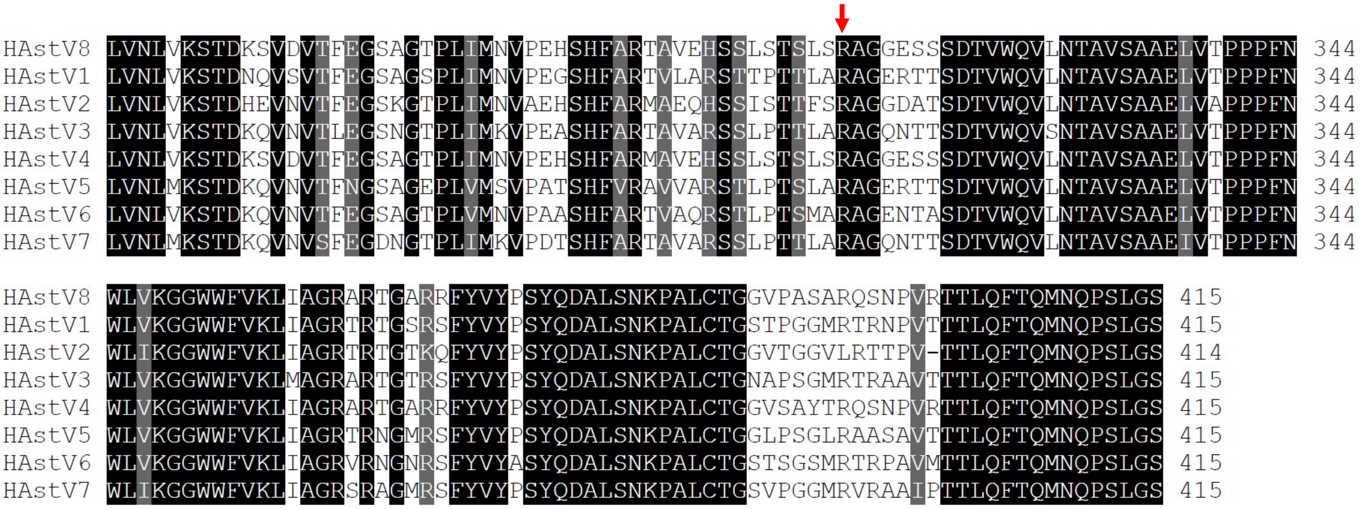

Figure S5. CP sequence alignment of all 8 classical HAstV strains. The P1 region (aa264-415) is shown. Strictly conserved and homologous residues are in black and grey shades, respectively. R313 is highlighted by a red arrow.

```
HAstV8     LVNLVK-----STDKSVDVTFEGSAGTPLIMNVPEHSHFARTAVEHSSLSTSLSRAGGESSSDTVWQVLNTAVSAAELVT 339
HAstV1     LVNLVK-----STDNQVSVTFEGSAGSPLIMNVPEGSHFARTVLARSTTPTTLARAGERTTSDTVWQVLNTAVSAAELVT 339
HAstV2     LVNLVK-----STDHEVNVTFEGSKGTPLIMNVAEHSHFARMAEQHSSISTTFSRAGGDATSDTVWQVLNTAVSAAELVA 339
HAstV3     LVNLVK-----STDKQVNVTLEGSNGTPLIMKVPEASHFARTAVARSSLPTTLARAGQNTTSDTVWQVSNTAVSAAELVT 339
HAstV4     LVNLVK-----STDKSVDVTFEGSAGTPLIMNVPEHSHFARMAVEHSSLSTSLSRAGGESSSDTVWQVLNTAVSAAELVT 339
HAstV5     LVNLMK-----STDKQVNVTFNGSAGEPLVMSVPATSHFVRAVVARSTLPTSLARAGERTTSDTVWQVLNTAVSAAELVT 339
HAstV6     LVNLVK-----STDKQVNVTFEGSAGTPLVMNVPAASHFARTVAQRSTLPTSMARAGENTASDTVWQVLNTAVSAAELVT 339
HAstV7     LVNLMK-----STDKQVNVSFEGDNGTPLIMKVPDTSHFARTAVARSSLPTTLARAGQNTTSDTVWQVLNTAVSAAEIVT 339
AstV VA1   LGMLER------VTETTNASIEVANGN-MIMTVPQNSQLARHMSERFERTTNASTVG-----ETIWQIVDEGAGLVANVA 332
AstV MLB2  LATLTQHKDDSETTKTLAISAEG-ANEPVEMLIPVGKWNMKE-----------QYGGNGTLGEAIYQVVDTSVTVVTDLF 332

HAstV8     PPPFNWLVKGGWWFVKLIAGRAR----TGARR------FYVYPSYQDALSNKPALCTGGVPASARQSNPVRTTLQFTQMN 409
HAstV1     PPPFNWLVKGGWWFVKLIAGRTR----TGSRS------FYVYPSYQDALSNKPALCTGSTPGGMRTRNPVTTTLQFTQMN 409
HAstV2     PPPFNWLIKGGWWFVKLIAGRTR----TGTKQ------FYVYPSYQDALSNKPALCTGGVTGGV-LRTTPVTTLQFTQMN 408
HAstV3     PPPFNWLVKGGWWFVKLMAGRAR----TGTRS------FYVYPSYQDALSNKPALCTGNAPSGMRTRAAVTTTLQFTQMN 409
HAstV4     PPPFNWLVKGGWWFVKLIAGRAR----TGARR------FYVYPSYQDALSNKPALCTGGVSAYTRQSNPVRTTLQFTQMN 409
HAstV5     PPPFNWLVKGGWWFVKLIAGRTR----NGMRS------FYVYPSYQDALSNKPALCTGGLPSGLRAASAVTTTLQFTQMN 409
HAstV6     PPPFNWLVKGGWWFVKLIAGRVR----NGNRS------FYVYASYQDALSNKPALCTGSTSGSMRTRPAVMTTLQFTQMN 409
HAstV7     PPPFNWLIKGGWWFVKLIAGRSR----AGMRS------FYVYPSYQDALSNKPALCTGSVPGGMRVRAAIPTTLQFTQMN 409
AstV VA1   PPPFTWLIKGGWWFVKKLLGRSA-------NTDVQ---YLVYASLADAQNNRP------VEAQNYTKVTRQTTLSSTQIN 396
AstV MLB2  PPPFSWLGKASWWFIRKVIGKSKTYTLTGTNQQITADVYRVYTSASAARDDKPAI-----SSTAHQAKNLTGSVVITQIS 407

HAstV8     QPSLGHG 416
HAstV1     QPSLGHG 416
HAstV2     QPSLGHG 415
HAstV3     QPSLGHG 416
HAstV4     QPSLGHG 416
HAstV5     QPSLGHG 416
HAstV6     QPSLGHG 416
HAstV7     QPSLGHG 416
AstV VA1   APNTGPN 403
AstV MLB2  TDNILN- 413
```

Figure S6. CP core sequence alignment of all 8 classical HAstV strains with AstV VA1 and MLB2. The P1 region (HAstV8 aa264-415) is shown. Strictly conserved and homologous residues are in black and grey shades, respectively. R313 in HAstV8 is highlighted by a red arrow.
